# Optimization of target grouping in distributive stereotactic radiosurgery using the excel evolutionary solver

**DOI:** 10.1002/acm2.14608

**Published:** 2024-12-20

**Authors:** Chester Ramsey, Samuel Gallemore, Joseph Bowling

**Affiliations:** ^1^ Thompson Cancer Survival Center Knoxville Tennessee USA; ^2^ Department of Nuclear Engineering Complex University of Tennessee Knoxville Tennessee USA; ^3^ Fort Sanders Regional Gamma Knife Center Knoxville Tennessee USA

**Keywords:** distributive fractionation, Evolutionary Solver, GammaKnife, genetic algorithm, SRS

## Abstract

**Purpose:**

Distributive stereotactic radiosurgery (dSRS) is a form of fractionation where groups of metastases are treated with a full single‐fraction dose on different days. The challenge with dSRS is determining optimal target groupings to maximize the distance between targets treated in the same fraction. This study aimed to develop and validate an accessible optimization technique for distributing brain metastases into optimal treatment fractions using a genetic algorithm.

**Methods:**

The Evolutionary Solver in Excel was used to optimize the grouping of target volumes for distributive SRS fractionation. The algorithm's performance was tested using three geometric test cases with known optimal solutions, 400 simulations with randomly distributed target volumes, and clinical data from five GammaKnife patients. The objective function was defined as the sum of average distances between target volumes within each fraction, with constraints ensuring 2–5 targets per fraction, each target being assigned to only one fraction, and a constraint on the minimum distance between any two targets in the same fraction.

**Results:**

The Evolutionary Solver successfully identified optimal target groupings in all geometric test cases. Compared to random groupings, the mean distance between target volumes increased by 9%, from 68.1 ± 0.8  to 74.2 ± 1.1 mm post‐optimization, while the minimum distance between targets increased by 57%, from 24.9 ± 5.9  to 39.1 ± 7.5 mm. In clinical test cases, the mean distances improved from 81.6 ± 11.9 mm for manual target grouping to 85.6 ± 14.5 mm for optimized target grouping. The minimum separation improved from 35.2 ± 14.5 mm with manual grouping to 51.6 ± 14.7 mm with optimized grouping, corresponding to a mean improvement of 16.4 ± 6.1 mm.

**Conclusion:**

The Evolutionary Solver in Excel provides a systematic and reproducible method for optimizing distributive target groupings in SRS and enhances spatial separation.

## 1 INTRODUCTION

Historically, stereotactic radiosurgery (SRS) in the management of brain metastases (BMs) has been limited to the treatment of a limited number of targets, often 3–4 individual BMs where the largest individual tumor dimension was no greater than 3 cm.^1^, ^2^, ^3^ According to ASTRO clinical practice guidelines, SRS is conditionally recommended for patients with an Eastern Cooperative Oncology Group (ECOG) performance status of 0–2 and 5–10 intact BMs.^4^ However, the actual number of BMs that can be safely treated could be substantially larger.^5^, 

A recent Monte Carlo in silico study sought to estimate the maximum number of BMs that could be treated using a GammaKnife without exceeding the normal brain tolerances associated with whole brain radiation therapy (WBRT).^5^ Simulated tumors of various sizes were randomly placed throughout a representative MR dataset until the brain mean dose was 3 Gy, which corresponds to a single fraction of WBRT. The study concluded that many tumors (12–13) could be treated per day for 10 days without exceeding the mean brain dose of a typical WBRT course.

The treatment of a large number of BMs can present multiple challenges, including (1) prolonged treatment times, (2) increased complexity in accurately targeting multiple lesions, and (3) possible increased radiation toxicity. Kelly first proposed a technique for “divide‐and‐conquer” in SRS.^6^ BMs were divided into two to five groups of targets, with different groups being treated on different days to the full dose. Kelly hypothesized that 50 or more targets could be treated with this technique by lowering the biological effective dose (BED) to surrounding normal tissues.

Implementing the “divide‐and‐conquer” concept, Chen et al. introduced the concept of distributive SRS (dSRS) on the GammaKnife Perfexion/Icon (Elekta AB, Stockholm, Sweden) for the treatment of multiple BMs.^7^ Distributive SRS fractionation was defined as a form of fractionation where groups of individual metastases are treated sequentially. Each individual metastasis is treated to the full dose with a single fraction. However, the metastases are divided into multiple groups that are spatially distant from each other.

**FIGURE 1:**
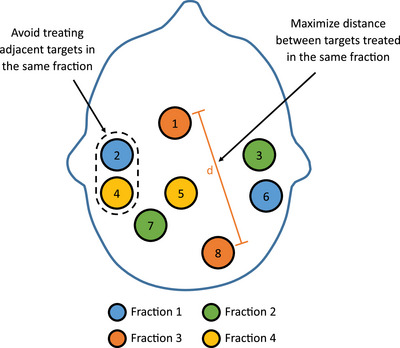
Example of target volume groupings for distributive SRS fractionation. Each individual target is only treated for one fraction.

Figure 1 shows an example of distributive SRS fractionation. In this example, targets 2 and 6 will be treated to the full prescribed dose for the first fraction. For the second fraction, targets 3 and 7 will be treated to the full prescribed dose. In fraction 3, targets 1 and 8 will be treated to the full prescribed dose. The remaining targets, 4 and 5, will be treated in fraction 4. In this example, each target would be treated with a single fraction SRS dose.

The challenge with distributive SRS fractionation is determining the optimal grouping of the targets. In the clinic, the grouping of targets in distributive SRS fractionation is typically determined manually by the treatment planning team by visual examination. The goal is to avoid treating adjacent targets on the same fraction while attempting to maximize the separation between targets that are treated in the same fraction. As the number of targets increases, this process becomes increasingly difficult.

In order to automate this process, Chen et al. utilized a technique that was modeled using electrostatic fields, where each target exerted a mutual repulsive force with all other targets.^7^ This approach reduces the potential energy of the BMs by separating them into different treatment fractions. While this model was effective and efficient in finding solutions, it requires the use of mixed‐integer quadratic programming, which may not be available in every clinic.

The purpose of this study was to develop a simple to implement optimization technique for separating BMs into distributive fraction groups. The goal was to develop a heuristic optimization technique that could be easily implemented using the Excel “Solver” Add‐In. Because Solver is included with most versions of Microsoft Excel, it is widely accessible and can be implemented in any clinic where Microsoft Excel is available.

## 2 MATERIALS AND METHODS

### 2.1 Evolutionary Solver algorithm

The Evolutionary Solver in Microsoft Excel's Solver Add‐in was employed to optimize the placement of target volumes for distributive fractionation.^8^ The Evolutionary Solver utilizes a genetic algorithm that simulates natural evolutionary processes to optimize solutions over successive generations. The refinement of solutions involves a series of genetic operations, including selection, crossover, and mutations that collectively evolve the population of solutions.

The genetic algorithm initiates the optimization with a random population of multiple solutions, with each individual (i.e., solution) evaluated based on an objective function value. Individuals (i.e., solutions) in the population with the highest objective functions are preserved from generation to generation, while solutions with the lowest objective functions are discarded. The individuals with the highest objective functions randomly trade chromosomes (i.e., variable values) to create new offspring in a process that mimics natural selection. In addition, the genetic algorithm incorporates random mutations in random chromosomes. This selection process progressively drives the population toward higher levels of fitness that is measured by a numerical objective function. The procedure continues until there is no significant improvement in the population or when specific user‐defined stopping conditions are met.

Users can influence the optimization process in the Evolutionary Solver by several adjustable parameters: population size, which can be set between 10 and 200 to define the number of elements per population; Random Seed, which initializes the pseudo‐random number generator used during the evolution (a zero value ensures a new random series for each run); mutation rate, controlling the frequency of mutations to enhance population diversity; Convergence, terminating the search when the top 99% of the population's objective function values change by less than the preset percentage; maximum time without improvement and maximum time, both of which define the duration of the search process. Table 1 shows the optimization options used in the Evolutionary Solver for this study.

**TABLE 1:** Optimization options used in the Evolutionary Solver.

Optimization options	Value
Convergence	0.0001
Mutation rate	0.075
Population size	100
Random seed	0
Max time (s)	120
Max time without improvement	40

### 2.2 Optimization objectives and constraints

The objective function for each individual in the population was related to the distance between the centers of each target volume, specified by the coordinate X (Left‐Right), Y (Anterior‐Posterior), and Z (Superior‐Inferior) in the stereotactic reference system. These distances (*d)* were calculated between each combination of target volumes assigned to a distributive group (i.e., fraction) using

(1) $$d=\sqrt{{\left(X_{2}-X_{1}\right)}^{2}+{\left(Y_{2}-Y_{1}\right)}^{2}+{\left(Z_{2}-Z_{1}\right)}^{2}}\;.$$

These distances were then used to calculate the average separation between target volumes for each distributive fraction. The objective function was the sum of the average separations for each distributive group. The goal of the optimization was to maximize the objective function, thereby achieving the greatest average distance between the individual target volumes within a single treatment fraction. The average distance between targets was selected as the objective function because it provides an intuitively clear and quantifiable metric for evaluating the spatial distribution of target volumes.

In addition to the objective function, the optimization was subject to a series of constraints designed to optimize the spatial distribution of the target volumes. Each distributed fraction was required to have between two and five individual target volumes. This constraint ensured that no fraction contained a single target volume for treatment delivery efficiency. Additionally, each target could only be assigned to one fraction to ensure a single‐fraction SRS dose was delivered to each target. The fraction number for each target was constrained to be an integer to eliminate the possibility of targets being assigned to partial fractions. Finally, a constraint on the minimum distance between targets was also utilized, which provided an adjustable parameter in the optimization.

### 2.3 Validation test patterns

**FIGURE 2:**
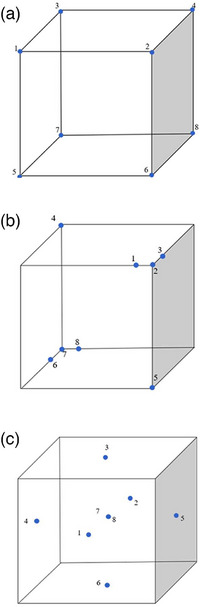
Geometric test cases with known optimal target grouping. Test 1 (a) consists of eight targets (blue circles) placed at the vertices of a cube. Test 2 (b) consists of eight total targets with three targets grouped in two of the vertices. Test 3 (c) consists of eight total targets, with one in the center of each face of the cube plus two targets in the center.

To test the Evolutionary Solver's ability to correctly distribute the target volumes, a series of three test cases were created using a cube‐based geometry. Targets were placed in the cube that had a known optimal distribution (Figure 2). The first test case consisted of a 10 × 10 × 10‐cm cube with the targets placed at each of the vertices. The optimal combination of target groupings for four fractions should consist of targets at opposing vertices. The second test case involved a cube with targets placed at opposing vertices. One pair of opposing vertices contained single targets, while another pair contained multiple closely grouped targets. The purpose of this test was to evaluate the Solver's ability to optimally separate targets that are tightly packed. The third test case included targets positioned at the centers of each face of the cube, along with two additional targets located within the interior of the cube.

### 2.4 Simulated target volumes

Simulated target volumes were randomly distributed throughout a representation of an adult brain using the random function in Excel, which is based on a Gaussian distribution. This approach facilitates the simulation of a significantly larger number of cases than are typically encountered in clinical settings providing a robust dataset for analysis.

For the purpose of this study, only the geometric centers of each target volume were simulated. This was based on the premise that only the coordinates of the center of each target are necessary for creating effective distributive groupings. To minimize data entry in the clinical setting, neither the volume of the target volumes or the dose distribution were incorporated into the optimization calculations.

A total of 400 simulations were performed for groupings of 8, 12, 16, and 20 target volumes. The group with eight target volumes was divided into three distributive fractions, the twelve‐target volume group into four, the sixteen into four as well, and the twenty‐target volume group into five distributive fractions. This simulation allowed for the evaluation of a wide range of distributive strategies and their potential improvements.

The maximum optimization time was set at 2 min per simulation, during which approximately 20 000 generations were typically produced. To evaluate the effectiveness of the optimization, the mean distance and the closest distance between target volumes were recorded both for the initial random distribution and for each subsequent simulation.

### 2.5 Clinical validation

The Evolutionary Solver was validated using previously treated GammaKnife Icon patients with greater than seven target volumes. The X (Left‐Right), Y (Anterior‐Posterior), and Z (Superior‐Inferior) coordinates for the center of each target volume were manually extracted from GammaPlan (Elekta AB, Stockholm, Sweden) by selecting the individual target volume and recording the stereotaxic reference coordinates. These values were entered into the Evolutionary Solver, which maximized the mean distance between target volumes.

The performance of the Evolutionary Solver was benchmarked against the manual assignment of target volumes into groups. The manual grouping was performed by an experienced treatment planner who used visual examination and clinical judgment to distribute the targets across multiple fractions. The mean distance between target volumes was calculated for the manual grouping and compared against the Evolutionary Solver.

## 3 RESULTS

### 3.1 Validation test patterns

A series of three geometric test cases were created using a 10 × 10 × 10 cm cube with predetermined optimal groupings in the form of target volume pairs. In each of these test scenarios, the Evolutionary Solver successfully identified the optimal configurations. Across all tests, the algorithm consistently determined that the best combinations for dividing the target volumes into four fractions involved pairing targets 1 & 8, 2 & 7, 3 & 6, and 4 & 5. This consistent identification of optimal groupings in each scenario shows the Evolutionary Solver's effectiveness and reliability in achieving optimal distributive optimization for simple geometries with known solutions.

### 3.2 Simulated target volumes

The algorithm was tested with simulated targets randomly placed within a representation of an adult brain. The results demonstrated an improvement in target volume separation. The mean distance between target volumes increased by 9% from 68.1 ± 0.8 mm (non‐optimized) to 74.2 ± 1.1 mm (optimized). The minimum distance between the closest target volumes increased by 57% from 24.9 ± 5.9 mm (non‐optimized) to 39.1 ± 7.5 mm (optimized). As shown in Table 2, the average improvement was 6.0 ± 1.2 mm in average distance between targets and 14.2 ± 2.1 mm in the minimum distance between targets.

**TABLE 2:** Results from testing the Evolutionary Solver for randomly positioned target volumes in a simulated head phantom

				Random target grouping		Evolutionary grouping		Improvement	
*N*	Total targets	Distributive groups	Targets per fraction	Average distance (mm)	Minimum separation (mm)	Average distance (mm)	Minimum separation (mm)	Average distance (mm)	Minimum separation (mm)
100	8	3	2.7	67.4	32.4	75.1	47.6	7.7	15.2
100	12	4	3.0	68.2	26.7	73.7	43.1	5.6	16.4
100	16	4	4.0	67.7	21.0	72.8	34.6	5.0	13.6
100	20	5	4.0	69.3	19.6	75.1	31.2	5.8	11.6

The average distance between target volumes and the minimum separation are shown for the initial random grouping and for the optimal distributive grouping after optimization with the Evolutionary Solver.

### 3.3 Clinical validation

The distributive SRS fractionation genetic algorithm was tested on five patients previously treated on the GammaKnife Icon with between 7 and 16 targets. The mean distances improved from 81.6 ± 11.9 mm for manual target grouping to 85.6 ± 14.5 mm for optimized target grouping (Table 3). The minimum separation improved from 35.2 ± 14.5 mm with manual grouping to 51.6 ± 14.7 mm with optimized grouping, corresponding to a mean improvement of 16.4 ± 6.1 mm.

**TABLE 3:** Improvements in target groupings for clinical cases using the Evolutionary Solver

				Manual target grouping		Evolutionary grouping		Improvement	
Patient	Total targets	Distributive groups	Targets per fraction	Average distance (mm)	Minimum separation (mm)	Average distance (mm)	Minimum separation (mm)	Average distance (mm)	Minimum separation (mm)
1	7	3	2.3	93.2	50.0	102.8	68.5	9.6	18.5
2	11	4	2.8	90.6	41.8	93.0	62.5	2.4	20.7
3	12	4	3.0	70.6	39.8	70.2	45.4	−0.4	5.6
4	12	4	3.0	86.3	32.3	91.1	50.2	4.8	17.9
5	16	4	4.0	67.1	11.9	70.7	31.3	3.6	19.4

The average distance between target volumes and the minimum separation are shown for manual grouping and for the optimal distributive grouping after optimization with the Evolutionary Solver.

## 4 DISCUSSION

Distributive SRS requires the assignment of target volumes into treatment groups. While manual assignment is the most common approach, automated optimization of the target groupings offers several advantages. Manual grouping of target volumes can vary significantly between users, leading to inconsistent and potentially suboptimal groupings. When compared to manual grouping, the minimum separation between target volumes was improved in all cases using the Evolutionary Solver. Grouping the targets using an optimization technique also provides a systematic and reproducible method that can reduce user‐dependent variability and ensure a more consistent approach to target grouping. This effect was observed in the improvement in minimum separation for the test patients (*n* = 5) and the simulated targets (*n* = 400), where the improvement in minimum separation was 16.4 ± 6.1  and 14.2 ± 2.1 mm, respectively.

Even though the improvements in average and minimum separations are on the order of millimeters, these improvements can significantly reduce the impact of dose overlap between targets treated in the same fraction. SRS inherently has steep dose gradients with a rapid falloff in dose. This dose gradient can be characterized using the dose fall‐off factor (Δr), which is defined as the linear distance from the edge of the PTV to the 50% isodose cloud.^9^ Previous studies have reported dose fall‐off factors for both linear accelerator‐based SRS and GammaKnife‐based SRS for a range of target volumes from 0.2 to 44 cm^3^.^9^, ^10^ Values of Δr for the Eclipse‐TrueBeam combination ranged from 2.2  to 6.4 mm, while Δr for the GammaKnife Icon ranged from 1.9 to 8.3 mm. Given this rapid falloff to the 50% isodose, even small improvements have the potential to reduce the integral healthy brain dose on a given day relative to less optimized grouping strategies.

It was observed that the Evolutionary Solver did not always result in an increase in the average separation between targets. For the clinical cases, the average separation between targets increased in four out of the five patients, with one patient having a decrease in average separation of 0.4 mm. In this case, the constraint on minimum distance between targets in the optimization algorithm resulted in an increase in the minimum separation of 5.6 mm. This resulted in an improvement in the overall grouping because the minimum is the most important metric, as it is the dose overlap from adjacent targets that distributive treatment is attempting to eliminate.

This study focused on the use of a heuristic approach in the form of a genetic algorithm to solve the SRS distributive grouping problem. Heuristic approaches have been used for decades to solve the “Traveling Salesman Problem (TSP),” where one tries to find the shortest route between locations that are visited once. In the case of distributive SRS, it is similar to an inverse TSP where the goal is to maximize instead of minimize the route. The use of a genetic algorithm to solve distributive SRS groupings is an efficient approach to optimization, with the limitation that the solution may not be the global optimum.

The primary advantage of the Evolutionary Solver is that it is easy to implement. The use of Microsoft Excel's Add‐in makes this approach widely accessible, as Excel is commonly available in clinical settings. This accessibility ensures that clinics can adopt the Evolutionary Solver without the need for specialized software or coding experience.

One limitation of the current approach is that it does not take dose distribution into consideration. This work focuses instead on maximizing the mean distance between target volumes, which is a geometric objective. This approach ensures optimal spatial separation but does not account for the actual distribution of radiation dose. Future work will aim to incorporate dosimetric factors into the optimization process by adding an additional constraint that maximizes the distance between the 50% isodose surface of the targets in each distributive grouping. Including this constraint will help ensure that the optimization not only separates targets spatially but also minimizes the overlap of high‐dose regions between targets within the same fraction, thereby minimizing radiation dose to healthy brain tissue.

While this study used GammaKnife cases for clinical validation, the distributive SRS optimization technique is not unique to GammaKnife. The Evolutionary Solver technique developed in this study can be applied to any SRS modality including single isocenter multiple target (SIMT) linac‐based treatments. The only input needed for the Evolutionary Solver is the DICOM coordinates of the target centers. Most planning systems provide tools within the contour workspace that automatically center the target in the axial, coronal, and sagittal views, displaying the DICOM coordinates directly. This makes it straightforward for users to read and enter these coordinates into the optimization spreadsheet. This step can be performed using only the target contours before planning, enabling the creation of individual plans for each distributive group.

## 5 CONCLUSION

A genetic algorithm was used to optimize the grouping of target volumes for distributive SRS fractionation. The algorithm was successfully validated using test patterns, randomly generated targets, and patient data. The validation demonstrated that the algorithm effectively increases both the mean and the minimum distance between target volumes, thereby enhancing the spatial separation within treatment groups. This approach provides a systematic, reproducible method for target grouping that reduces user‐dependent variability. The use of Microsoft Excel's Evolutionary Solver Add‐in makes this technique widely accessible and easy to implement in clinical settings, facilitating its adoption across various SRS modalities.

## AUTHOR CONTRIBUTIONS

Chester Ramsey—Conception and design of study, data analysis and interpretation, manuscript drafting. Samuel Gallemore—Data acquisition, initial data analysis. Joseph Bowling—Creation of original clinical plans used in the study, data interpretation. All authors participated in manuscript revisions.

## CONFLICT OF INTEREST STATEMENT

The authors declare no conflicts of interest.

## Supporting information



Supporting information

## Data Availability

The data that support the findings of this study are available from the corresponding author upon reasonable request.
